# A preliminary study of skin ultrasound in diffuse cutaneous systemic sclerosis: Does skin echogenicity matter?

**DOI:** 10.1371/journal.pone.0174481

**Published:** 2017-03-24

**Authors:** He Liu, Yong Hou, Qing-li Zhu, Dong Xu, Liang Wang, Jian-chu Li, Yu-xin Jiang, Qian Wang, Meng-tao Li, Feng-chun Zhang, Xiao-feng Zeng

**Affiliations:** 1 Department of Ultrasound, Peking Union Medical College Hospital, Chinese Academy of Medical Sciences & Peking Union Medical College, Beijing, China; 2 Department of Rheumatology and Clinical Immunology, Peking Union Medical College Hospital, Chinese Academy of Medical Sciences & Peking Union Medical College, Beijing, China; Keio University, JAPAN

## Abstract

**Objective:**

To evaluate the usefulness of skin ultrasound and acoustic radiation force impulse (ARFI) quantification in diffuse cutaneous systemic sclerosis (dcSSc).

**Patients and methods:**

28 patients with dcSSc, and 15 age gender matched normal controls were recruited. Skin echogenicity, thickness, and ARFI quantification were measured by ultrasound at 17 sites corresponding to the modified Rodnan skin score (mRSS) in each participant. Compared with controls, skin echogenicity of dcSSc patients was classified into isoechoic, hypoechoic, and hyperechoic. The skin thickness, ARFI quantification and mRSS were compared between isoechoic, hypoechoic, hyperechoic and controls.

**Results:**

In patients with dcSSc, the skin thickness increased as the echogenicity changed on the order of isoechoic, hypoechoic and hyperechoic. ARFI quantification was significantly higher in hyperechoic than isoechoic (p<0.001). The mRSS were significantly higher in hyperechoic and/or hypoechoic than isoechoic. For isoechoic patients and healthy controls, the skin echogenicity or thickness was no significant different, however, the ARFI quantification was significantly higher in isoechoic than controls.

**Conclusion:**

Skin ultrasound is feasible for assessing the skin involvement in dcSSc. Skin echogenicity correlates with skin thickness, stiffness, and mRSS. ARFI quantification may be more sensitive to detect skin changes, compared with skin echogenicity and thickness.

## Introduction

Systemic sclerosis (SSc) is a heterogeneous autoimmune disorder of unknown aetiology, which may involve the skin and internal organs to varying degrees and with different courses. The extent of skin involvement correlates with survival and prognosis [1, 2]. Ultrasound has been demonstrated to be a reliable tool to measure skin thickness, echogenicity, and stiffness, and it is increasing employed in SSc. Skin thickness increased in SSc patients compared with healthy controls [3], and was significant different between edematous, fibrotic, and atrophic phases [4, 5]. Echographic images of SSc patients skin differed from healthy controls [6]. The degree of skin echogenicity correlated with the amount of sclerosis on histologic examination [7]. During the oedematous phase, skin echogenicity was low and skin thickness was high. When the oedematous phase was replaced by the indurative phase, skin echogenicity increased and skin thickness decreased [8]. Skin stiffness of SSc patients is more recent and less investigated [9–11]. The principle of this technique is the reduced skin elasticity caused by excessive dermal deposition of collagenous and non-collagenous extracellular matrix mediated fibrosis [12]. Increased skin stiffness, shown by specific color or higher ARFI quantification, was reported in SSc patients compared with healthy controls [9–11]. To our knowledge, the correlations between ultrasound measured skin thickness, echogenicty, and stiffness have not been fully conducted. The purpose of this study was therefore to investigate the correlations between them, and explore the usefulness of ultrasound, with the main focus on skin echogenicity and stiffness, in SSc patients.

## Patients and methods

### Patients

28 patients with dcSSc and 15 age gender matched controls were prospectively recruited from the rheumatology department of Peking union medical college hospital. The patients, who all met the American College of Rheumatology 1980 criteria [13] or American College of Rheumatology/European League Against Rheumatism 2013 criteria [14] for the classification of scleroderma and dcSSc [15], underwent clinical and serological assessment at entry. The disease duration was calculated from the onset of Raynaud’s phenomenon. An experienced physician assigned each patient mRSS on 0–3 ordinal scale over 17 anatomical sites according to Moore et al seventeen point dermal ultrasound scoring system [16]. The physician was trained at the European League Against Rheumatism Scleroderma Trials and Research group course and blinded to the result of the ultrasound assessment. The 17 sites were bilateral middle finger, hand dorsum, forearm, upper arm, thigh, lower leg, foot dorsum, forehead, anterior chest, and anterior abdomen. The ethics committee of Peking Union Medical College Hospital approved the study (No. S-191), and all participants signed informed consent.

### Ultrasound examination and image analysis

**Fig 1:**
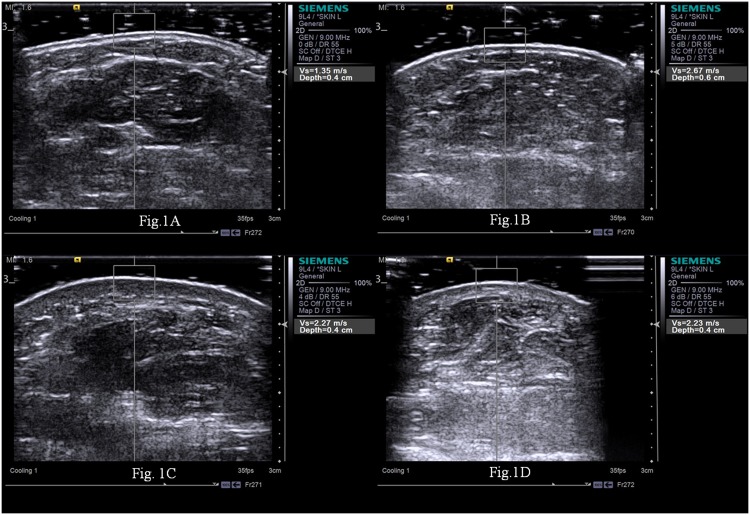
Ultrasound findings of the dcSSc patients and the control’s right forearm skin. The echogenicity was determined as compared with site-matched, normal skin in healthy controls (Fig 1A), and was classified into isoechogenic (Fig 1B), hypoechogenic (Fig 1C), and hyperechogenic (Fig 1D).

**Fig 2:**
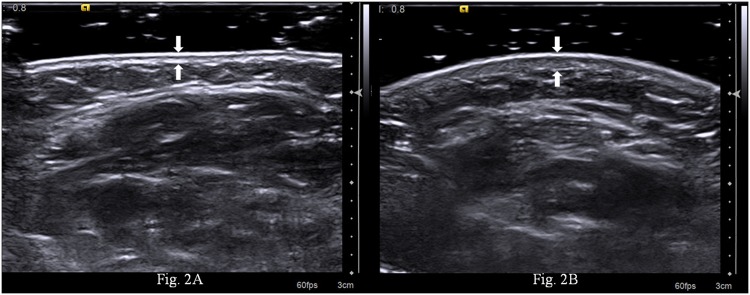
Ultrasound measured skin thickness of the dcSSc patients and the control’s right forearm. The skin thickness was 0.6mm (between arrows) for controls (Fig 2A), and 1.0mm (between arrows) for patients (Fig 2B).

Ultrasound and ARFI quantification were performed over the 17 sites corresponding to mRSS by an experienced ultrasound physician, who was engaged in superficial organ examination for more than 18 years and unaware of the patients’ clinical data. A Siemens S2000 ultrasound system (S2000; Siemens Medical Solutions, Inc. Siemens Healthcare, Erlangen, Germany) fitted with a 9L4 MHz linear probe was used. To increase the accuracy of the measurements, the probe was placed perpendicularly to the skin, and a layer of gel was applied to minimize the compression from the transducer to the skin. The ultrasound image was adopted when the epidermis, dermis, and subcutis were clearly visualized and the interfaces between them were parallel and distinct. The skin stiffness was measured by ARFI quantification. With ARFI technique in the form of Virtual Touch Quantification (VTQ), transducers are used to mechanically excite tissue with short-duration acoustic radiation forces, leading to shear waves propagation away from the region of excitation [9, 17]. The stiffer the tissue, the faster the shear waves propagate. Thus, the shear wave velocity measured by ARFI quantification gives tissue stiffness information of the region of interest (ROI). The ROI box had a preset size of 6mm (width) × 5mm (depth). Five trials of ARFI quantification was performed at each site. When “X” displayed on the screen, the measurements were interpreted as invalid. The five consecutive ARFI quantification measurements with no “X” were taken and the results were averaged. All static ultrasound images were stored and later analyzed by the other three independent outside ultrasound physicians with more than 20 years experience in superficial organs examination. Two ultrasound physicians evaluated all images independently and blinded to the patients’ clinical information. In cases in which there was a discrepancy between the two reviewers, a third ultrasound physician served as a blinded expert. They discussed to reach a consensus for classifying the patient’s skin echogenicity. The patient’s skin echogenicity of each site was compared with site matched normal skin in healthy controls (Fig 1A), and classified into isoechogenic (Fig 1B), hypoechogenic (Fig 1C), and hyperechogenic (Fig 1D). The skin thickness, the combined epidermis and dermis, was determined (Fig 2).

### Statistical analysis

SPSS software version 14.0 was used for statistical analysis, with p<0.05 considered statistically significant. Data were expressed as median (lower quartile, upper quartile). The intraclass correlation coefficient (ICC) was calculated to examine the inter-observer reliability of skin echogenicity classification. Differences in skin thickness, ARFI quantification and mRSS between isoechogenic, hypoechogenic, hyperechogenic and controls were assessed by Kruskal-Wallis test with Bonferroni multiple testing correction.

## Results

**Fig 3:**
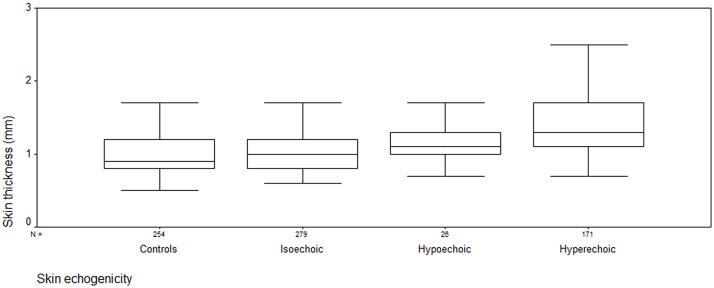
The skin thickness of dcSSc patients and controls. Box plots represent the 25 and 75th percentile of measures, the median (line in box) and the minimum and maximum values (whiskers). The thickness increased as the echogenicity changed on the order of isoechoic, hypoechoic and hyperechoic (p<0.001). No significant difference was found between isoechoic patients and healthy controls (p = 0.142).

**Fig 4:**
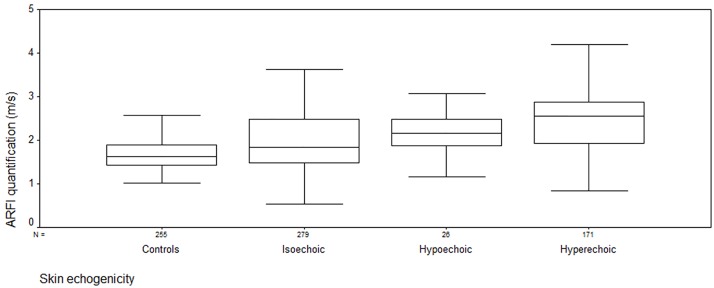
The ARFI quantification of dcSSc patients and controls. Box plots represent the 25 and 75th percentile of measures, the median (line in box) and the minimum and maximum values (whiskers). The ARFI quantification was significantly higher in hyperechoic than isoechoic (p<0.001), no significant difference was found between hyperechoic and hypoechoic, or between hypoechoic and isoechoic (p = 0.117). The ARFI quantification was significantly higher in isoechoic than controls (p<0.001).

Table 1 showed the clinical data of the patients. Table 2 showed the skin thickness, ARFI, and mRSS of dcSSc patients and controls. ICC of two reviewers’ skin echogenicity classification was 0.608 (p<0.001). In patients with dcSSc, the skin thickness was 1.0 (0.8, 1.2) mm, 1.1 (1.0, 1.3) mm, 1.3 (1.1, 1.7) mm for isoechoic, hypoechoic and hyperechoic respectively. The thickness increased as the echogenicity changed on the order of isoechoic, hypoechoic and hyperechoic (p<0.001) (Fig 3). ARFI quantification was 1.83 (1.48, 2.48) m/s, 2.16 (1.86, 2.49) m/s, 2.55 (1.92, 2.88) m/s for isoechoic, hypoechoic and hyperechoic respectively. The quantification was significantly higher in hyperechoic than isoechoic (p<0.001), no significant difference was found between hyperechoic and hypoechoic, or between hypoechoic and isoechoic (p = 0.117) (Fig 4). The local mRSS was expressed as median (lower quartile, upper quartile) for the three groups (isoechoic, hypoechoic and hyperechoic) respectively, and it was 0 (0, 1), 1 (0, 1), 1(0, 2) for isoechoic, hypoechoic and hyperechoic respectively. The mRSS was significantly higher in hyperechoic and/or hypoechoic than isoechoic (p<0.001), no significant difference was found between hyperechoic and hypoechoic (p = 0.600).

**Table 1:** Clinical data of 28 patients with dcSSc.

Characteristic	Patients	Controls
Age (years)	50.5 (19–65)	43 (21–72)
Sex, female: male	22: 6	13: 2
Disease duration, median (range) months	36 (4–204)	
Anti-topoisomerase antibodies(+/−)	17/11	
Anti-centromere antibodies(+/−)	0/28	
ANA(+/−)	26/2	
mRSS, median (range)	10 (4–23)	

**Table 2:** Skin thickness, ARFI, and mRSS of dcSSc patients and controls.

	Patients			Controls	
Site	Skin thickness(mm)	ARFI(m/s)	mRSS	Skin thickness(mm)	ARFI(m/s)
Right middle finger	1.1(1.0,1.3)	2.1(1.35,2.58)	1(0,1)	0.6(0.4,0.8)	1.0(0.8,1.34)
Right hand dorsum	1.2(1.0,1.6)	2.5(1.83,2.83)	1(1,2)	0.6(0.5,0.8)	1.0(0.8,1.28)
Right forearm	1.2(0.9,1.4)	2.6(2.3,2.89)	1(1,2)	0.6(0.4,0.7)	0.8(0.7,1.1)
Right upper arm	0.9(0.7,1.1)	1.57(1.35,1.87)	1(0,1)	0.5(0.5,0.8)	0.9(0.8,1.42)
Left middle finger	1.1(1.0,1.2)	2.49(1.91,2.81)	1(0,1)	0.5(0.4,0.7)	1.0(0.7,1.44)
Left hand dorsum	1.2(1.1,1.3)	2.4(1.8,2.84)	1(1,2)	0.7(0.4,0.8)	1.0(0.8,1.19)
Left forearm	1.1(0.9,1.4)	2.55(2.26,2.88)	1(1,2)	0.6(0.5,0.7)	1.0(0.8,1.48)
Left upper arm	0.9(0.8,1.2)	1.47(1.32,1.83)	1(0,1)	0.5(0.4,0.6)	1.0(0.8,1.41)
Forehead	0.9(0.8,1.1)	1.59(1.33,1.97)	1(1,1)	0.9(0.6,1.0)	1.2(0.9,1.38)
Anterior chest	1.4(1.1,1.6)	2.56(1.84,2.97)	1(0,1)	1.1(0.8,1.4)	1.4(1.3,1.59)
Anterior abdomen	1.5(1.1,2.1)	2.32(1.47,2.73)	0(0,1)	1.5(1.1,2.0)	1.71(1.4,2.0)
Right thigh	1.2(1.0,1.6)	2.21(1.8,2.64)	0(0,0)	1.0(0.6,1.4)	1.6(0.8,1.9)
Right lower leg	1.1(1.0,1.3)	2.02(1.63,2.55)	0(0,0)	0.8(0.6,1.0)	1.4(1.0,1.5)
Right foot dorsum	0.8(0.7,1.3)	1.91(1.33,2.46)	0(0,0)	0.5(0.4,0.7)	0.6(0.8,1.24)
Left thigh	1.2(1.1,1.7)	2.15(1.85,2.72)	0(0,0)	1.0(0.8,1.5)	1.4(1.1,1.9)
Left lower leg	1.1(0.9,1.3)	2.25(1.82,2.69)	0(0,0)	0.8(0.6,1.1)	1.2(1.0,1.5)
Left foot dorsum	0.8(0.7,1.1)	1.54(1.34,1.92)	0(0,0)	0.5(0.4,0.6)	0.8(0.7,1.45)

Between isoechoic patients and healthy controls, the skin thickness was no significant different (1.0 (0.8, 1.2) mm *v*.*s*. 0.7(0.5, 0.9) mm, p = 0.142), however, the ARFI quantification was significantly higher in isoechoic than controls (1.83(1.48, 2.48) m/s *v*.*s*. 1.1(0.8, 1.49) m/s, p<0.001).

## Discussion

Our study showed that skin echogenicity correlated with skin thickness and local mRSS. The thickness increased as the echogenicity changed on the order of isoechoic, hypoechoic and hyperechoic. The mRSS was significantly higher in hyperechoic and/or hypoechoic than isoechoic. We hypothesized that isoechogenicity in our study may be associated with very early disease or atrophy, while hypoechogenicity and hyperechogenicity may correlate with edema and sclerosis. Our results indicate that ultrasound measured skin thickness is more sensitive than mRSS to detect skin changes, which is in accordance with previous studies [2, 18, 19]. Ultrasound role is becoming more and more relevant in systemic sclerosis. The majority of articles reported the use of thickness for skin involvement assessment, only a few studied skin echogenicity [3, 4, 7, 8, 12]. Hesselstrand et al [8] reported the inverse relationship between skin echogenicity and thickness in SSc patients with short disease duration (<2 years). Over the five examined sites (finger, hand dorsum, forearm, leg, and chest), Hesselstrand et al found a mild-to-moderate positive correlation between local skin thickness and the local/total mRSS (spearman correlation coefficient +0.36 ~ +0.72), while a mild negative correlation between local skin echogenicity and the local/total mRSS (spearman correlation coefficient -0.18 ~ -0.47). Hesselstrand et al [8] used an arbitrary score of 0–255 to measure the mean echogenicity of a selected region. A low value represents high water content and a high value represents low water content. It’s difficult to obtain site-matched, unaffected skin for comparison in patients with dcSSc. Therefore, we determined echogenicity compared to site-matched normal skin in healthy controls. The theoretical basis for this is the previous findings that skin echo structure of SSc patients differed from healthy controls [6], and skin thickness and echogenicity was no significant different in healthy controls with regards to gender (male and female) and age (15–70 years old), except anatomical sites [20, 21].

Imaging of the elastic properties of skin using ARFI quantification has become the subject of increasing research in patients with SSc [10, 11]. Our pilot article showed increased ARFI quantification in dcSSc patients compared with healthy controls, and ARFI quantification was more sensitive than mRSS. In this study ARFI quantification was significantly higher in hyperechoic than isoechoic, suggesting higher grades of fibrosis in hyperechoic lesions. Moreover, ARFI quantification, instead of skin echogenicity or thickness, was significant different between isoechoic and controls, indicating ARFI quantification may be more sensitive than skin echogenicity and thickness to detect subtle skin changes. The results are similar to Kissin et al [18] and our previous study that demonstrated higher skin stiffness in so-called uninvolved skin as compared with healthy control skin, due to the abnormal endothelial activation and procollagen production [8, 22].

Our study is limited by the relatively small number of patients and controls included, single centre design and lack of information about ultrasound validity to detect change in patients’ follow up and clinical trial. These preliminary findings need to be confirmed in large studies.

In conclusion, skin ultrasound is feasible for assessing the skin involvement in dcSSc. Skin echogenicity correlates with skin thickness, stiffness, and mRSS. ARFI quantification may be more sensitive to detect skin changes, compared with skin echogenicity and thickness.

## Supporting information

S1 File The Institutional Review Board 1 approval. (JPG)

S2 File The Institutional Review Board 2 approval. (PDF)

S3 File STROBE checklist. (DOC)

S4 File Patients and controls data. (XLSX)
