# Supplementary figures and images for: A preliminary study of skin ultrasound in diffuse cutaneous systemic sclerosis: Does skin echogenicity matter?
Source: PLoS One. 2017 Mar 24;12(3):e0174481. doi: 10.1371/journal.pone.0174481 (PMC5365121; doi:10.1371/journal.pone.0174481)

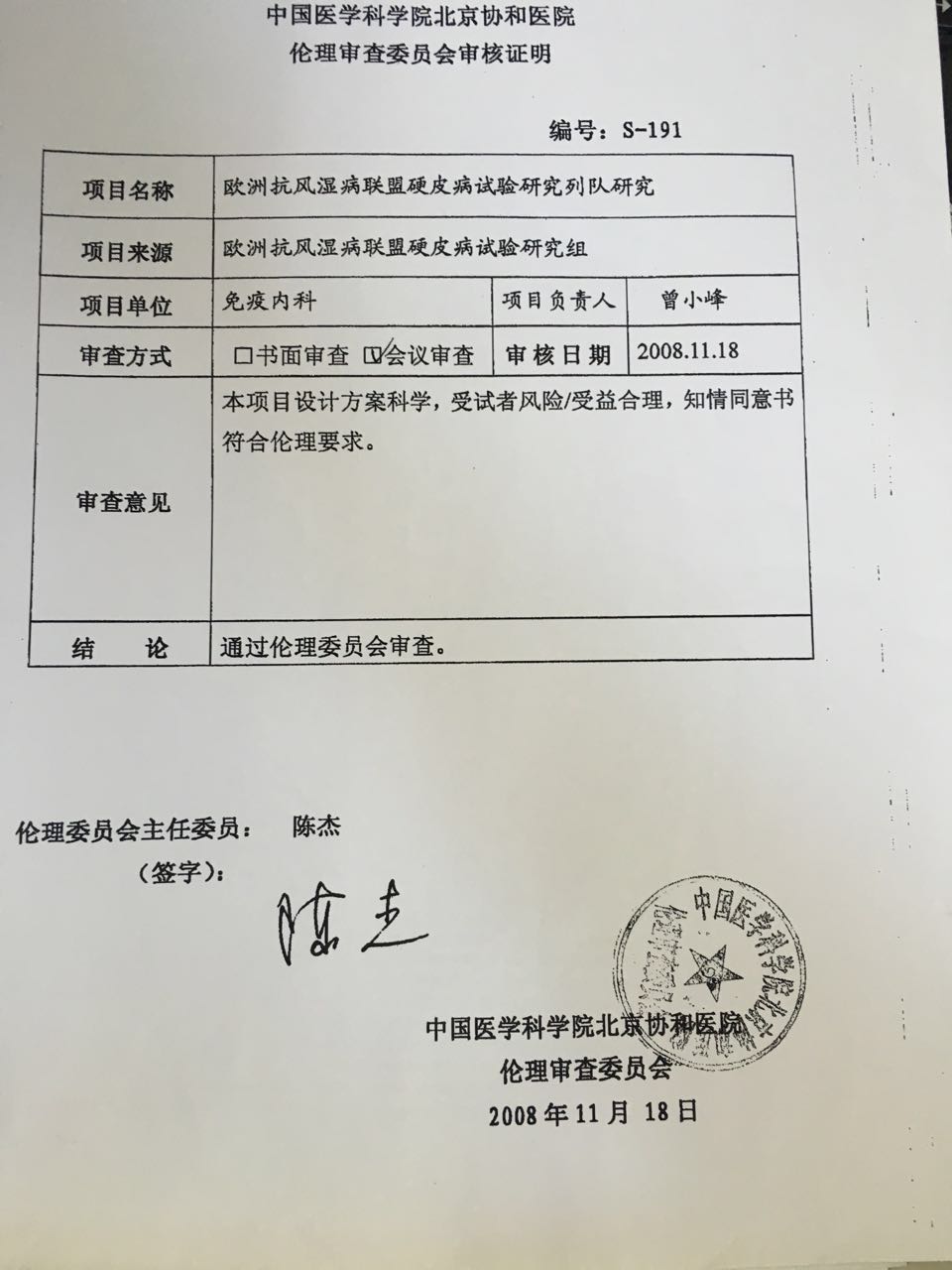

Supplement: S1 File — (JPG) [file pone.0174481.s001.JPG]
