# Supplementary material for: A preliminary study of skin ultrasound in diffuse cutaneous systemic sclerosis: Does skin echogenicity matter?
Source: PLoS One. 2017 Mar 24;12(3):e0174481. doi: 10.1371/journal.pone.0174481 (PMC5365121; doi:10.1371/journal.pone.0174481)
Supplement: S3 File — (DOC) [file pone.0174481.s003.doc]

STROBE Statement—checklist of items that should be included in reports of observational studies

|  | Item No. | Recommendation | Page  No. | Relevant text from manuscript |
| --- | --- | --- | --- | --- |
| **Title and abstract** | 1 | (*a*) Indicate the study’s design with a commonly used term in the title or the abstract | 1 | skin ultrasound in diffuse cutaneous systemic sclerosis |
|  |  | (*b*) Provide in the abstract an informative and balanced summary of what was done and what was found | 2-3 | skin ultrasound was performed in patients with diffuse cutaneous systemic sclerosis.  Skin echogenicity correlates with skin thickness, hardness, mRSS, and provides more information. ARFI quantification may be more sensitive to detect skin changes, compared with skin echogenicity and thickness. |
| Introduction | | | |  |
| Background/rationale | 2 | Explain the scientific background and rationale for the investigation being reported | 3 | To our knowledge, the correlations between ultrasound measured skin thickness, echogenicty, and hardness have not been fully conducted. |
| Objectives | 3 | State specific objectives, including any prespecified hypotheses | 4 | To investigate the correlations between skin thickness,echogenicity, and harness, and explore the usefulness of ultrasound, with the main focus on skin echogenicity and hardness, in SSc patients. |
| Methods | | | |  |
| Study design | 4 | Present key elements of study design early in the paper | 5 | Correlations between skin thickness,echogenicity, and hardness in dcSSc patients, with the focus of skin echogenicity and hardness. |
| Setting | 5 | Describe the setting, locations, and relevant dates, including periods of recruitment, exposure, follow-up, and data collection | 4 | 28 patients with dcSSc and 15 controls were prospectively recruited from the rheumatology department o Peking union medical college hospital. |
| Participants | 6 | (*a*) *Cohort study*—Give the eligibility criteria, and the sources and methods of selection of participants. Describe methods of follow-up  *Case-control study*—Give the eligibility criteria, and the sources and methods of case ascertainment and control selection. Give the rationale for the choice of cases and controls  *Cross-sectional study*—Give the eligibility criteria, and the sources and methods of selection of participants | 4 | 28 patients with dcSSc and 15 age gender matched controls were prospectively recruited from the rheumatology department of Peking union medical college hospital. The patients, who all met the American College of Rheumatology 1980 criteria or American College of Rheumatology/European League Against Rheumatism 2013 criteria for the classification of scleroderma and dcSSc. |
|  |  | (*b*) *Cohort study*—For matched studies, give matching criteria and number of exposed and unexposed  *Case-control study*—For matched studies, give matching criteria and the number of controls per case | 4 | Not applicable |
| Variables | 7 | Clearly define all outcomes, exposures, predictors, potential confounders, and effect modifiers. Give diagnostic criteria, if applicable | 2 | In patients with dcSSc, the skin thickness increased as the echogenicity changed on the order of isoechoic, hypoechoic and hyperechoic. ARFI quantification was significantly higher in hyperechoic than isoechoic (p＜0.001). MRSS were significantly higher in hyperechoic and/or hypoechoic than isoechoic. For isoechoic patients and healthy controls, the skin echogenicity or thickness was no significant different, however, the ARFI quantification was significantly higher in isoechoic than controls. |
| Data sources/ measurement | 8* | For each variable of interest, give sources of data and details of methods of assessment (measurement). Describe comparability of assessment methods if there is more than one group | 5-6 | The skin hardness was measured by ARFI quantification. Five trials of ARFI quantification was performed at each site. When “X” displayed on the screen, the measurements were interpreted as invalid. The five consecutive ARFI quantification measurements with no “X” were taken and the results were averaged. All ultrasound images were analyzed by the other three independent outside ultrasound physicians with more than 20 years experience in superficial organs examination. They discussed to reach a consensus when they disagreed with each other. The skin thickness, the combined epidermis and dermis, was determined. The echogenicity was classified as compared with site-matched, normal skin in healthy controls into isoechogenic, hypoechogenic, and hyperechogenic. |
| Bias | 9 | Describe any efforts to address potential sources of bias | 4 | Consecutive, blind |
| Study size | 10 | Explain how the study size was arrived at | 4 | 28 patients with dcSSc and 15 age gender matched controls were prospectively recruited. |

Continued on next page

| Quantitative variables | 11 | Explain how quantitative variables were handled in the analyses. If applicable, describe which groupings were chosen and why | 6 | Differences in skin thickness, ARFI quantification and mRSS between isoechogenic, hypoechogenic, hyperechogenic and controls were assessed by Kruskal-Wallis test with Bonferroni multiple testing correction. |
| --- | --- | --- | --- | --- |
| Statistical methods | 12 | (*a*) Describe all statistical methods, including those used to control for confounding | 6 | SPSS software version 14.0 (SPSS) was used for statistical analysis, with  p<0.05 considered statistically significant. Data were expressed as median (lower quartile, upper quartile). Differences in skin thickness, ARFI quantification and mRSS between isoechogenic, hypoechogenic, hyperechogenic and controls were assessed by Kruskal-Wallis test with Bonferroni multiple testing correction. |
|  |  | (*b*) Describe any methods used to examine subgroups and interactions | 6 | Kruskal-Wallis test with Bonferroni multiple testing correction |
|  |  | (*c*) Explain how missing data were addressed | 6 | No missing data |
|  |  | (*d*) *Cohort study*—If applicable, explain how loss to follow-up was addressed  *Case-control study*—If applicable, explain how matching of cases and controls was addressed  *Cross-sectional study*—If applicable, describe analytical methods taking account of sampling strategy | 4 | 28 patients with dcSSc and 15 age gender matched controls |
|  |  | (*e*) Describe any sensitivity analyses | 6 | Not applicable |
| Results | | | | |
| Participants | 13* | (a) Report numbers of individuals at each stage of study—eg numbers potentially eligible, examined for eligibility, confirmed eligible, included in the study, completing follow-up, and analysed | 4 | 28 patients with dcSSc and 15 age gender matched controls were prospectively recruited from the rheumatology department of Peking union medical college hospital. The patients, who all met the American College of Rheumatology 1980 criteria or American College of Rheumatology/European League Against Rheumatism 2013 criteria for the classification of scleroderma and dcSSc. |
|  |  | (b) Give reasons for non-participation at each stage | 4 | Not applicable |
|  |  | (c) Consider use of a flow diagram | 4 | Not applicable |
| Descriptive data | 14* | (a) Give characteristics of study participants (eg demographic, clinical, social) and information on exposures and potential confounders | 4 | 28 patients with dcSSc and 15 age gender matched controls were prospectively recruited from the rheumatology department of Peking union medical college hospital. The patients, who all met the American College of Rheumatology 1980 criteria or American College of Rheumatology/European League Against Rheumatism 2013 criteria for the classification of scleroderma and dcSSc. |
|  |  | (b) Indicate number of participants with missing data for each variable of interest | 4 | Not applicable |
|  |  | (c) *Cohort study*—Summarise follow-up time (eg, average and total amount) | 4 | Not applicable |
| Outcome data | 15* | *Cohort study*—Report numbers of outcome events or summary measures over time |  |  |
|  |  | *Case-control study—*Report numbers in each exposure category, or summary measures of exposure | 2-3 | skin ultrasound was performed in patients with diffuse cutaneous systemic sclerosis.  Skin echogenicity correlates with skin thickness, hardness, mRSS, and provides more information. ARFI quantification may be more sensitive to detect skin changes, compared with skin echogenicity and thickness. |
|  |  | *Cross-sectional study—*Report numbers of outcome events or summary measures |  |  |
| Main results | 16 | (*a*) Give unadjusted estimates and, if applicable, confounder-adjusted estimates and their precision (eg, 95% confidence interval). Make clear which confounders were adjusted for and why they were included | 2-3 | skin ultrasound was performed in patients with diffuse cutaneous systemic sclerosis.  Skin echogenicity correlates with skin thickness, hardness, mRSS, and provides more information. ARFI quantification may be more sensitive to detect skin changes, compared with skin echogenicity and thickness. |
|  |  | (*b*) Report category boundaries when continuous variables were categorized | 2-3 | Not applicable |
|  |  | (*c*) If relevant, consider translating estimates of relative risk into absolute risk for a meaningful time period | 2-3 | Not applicable |

Continued on next page

| Other analyses | 17 | Report other analyses done—eg analyses of subgroups and interactions, and sensitivity analyses | 2-3 | skin ultrasound was performed in patients with diffuse cutaneous systemic sclerosis.  Skin echogenicity correlates with skin thickness, hardness, mRSS, and provides more information. ARFI quantification may be more sensitive to detect skin changes, compared with skin echogenicity and thickness. |
| --- | --- | --- | --- | --- |
| Discussion | | | | |
| Key results | 18 | Summarise key results with reference to study objectives | 2-3 | skin ultrasound was performed in patients with diffuse cutaneous systemic sclerosis.  Skin echogenicity correlates with skin thickness, hardness, mRSS, and provides more information. ARFI quantification may be more sensitive to detect skin changes, compared with skin echogenicity and thickness. |
| Limitations | 19 | Discuss limitations of the study, taking into account sources of potential bias or imprecision. Discuss both direction and magnitude of any potential bias | 10 | Our study is limited by the relatively small number of patients and controls included, single centre design and lack of information about ultrasound validity to detect change in patients’ follow up and clinical trial. These preliminary findings need to be confirmed in large studies |
| Interpretation | 20 | Give a cautious overall interpretation of results considering objectives, limitations, multiplicity of analyses, results from similar studies, and other relevant evidence | 10 | skin ultrasound is feasible for assessing the skin involvement in dcSSc. Skin echogenicity correlates with skin thickness, hardness, mRSS and provides more information. ARFI quantification may be more sensitive to detect skin changes, compared with skin echogenicity and thickness |
| Generalisability | 21 | Discuss the generalisability (external validity) of the study results | 10 | skin ultrasound is feasible for assessing the skin involvement in dcSSc. Skin echogenicity correlates with skin thickness, hardness, mRSS and provides more information. ARFI quantification may be more sensitive to detect skin changes, compared with skin echogenicity and thickness |
| Other information | |  | | |
| Funding | 22 | Give the source of funding and the role of the funders for the present study and, if applicable, for the original study on which the present article is based |  | Not applicable |

*Give information separately for cases and controls in case-control studies and, if applicable, for exposed and unexposed groups in cohort and cross-sectional studies.

**Note:** An Explanation and Elaboration article discusses each checklist item and gives methodological background and published examples of transparent reporting. The STROBE checklist is best used in conjunction with this article (freely available on the Web sites of PLoS Medicine at http://www.plosmedicine.org/, Annals of Internal Medicine at http://www.annals.org/, and Epidemiology at http://www.epidem.com/). Information on the STROBE Initiative is available at www.strobe-statement.org.
