# Supplementary material for: A preliminary study of skin ultrasound in diffuse cutaneous systemic sclerosis: Does skin echogenicity matter?
Source: PLoS One. 2017 Mar 24;12(3):e0174481. doi: 10.1371/journal.pone.0174481 (PMC5365121; doi:10.1371/journal.pone.0174481)
Supplement: S2 File — (PDF) [file pone.0174481.s002.pdf]

北京協和醫院
PEKING UNION MEDICAL COLLEGE HOSPITAL

PROTOCOL NUMBER: **S-191**

PROTOCOL TITLE: The European League Against Rheumatism (EULAR) Scleroderma Trial and Research group (EUSTAR) China cohort study

IRB APPROVAL DATE: **2008-11-18**

IRB APPROVAL TYPE:

Dear Dr. Zeng Xiao-feng:

The Institutional Review Board of Peking Union Medical College Hospital reviewed the above-named protocol. The project was scientific and logical; the participants' Risk/ Benefit Assessment was reasonable; the informed consent fulfilled the requirement of ethics. We approved the research.

Jie Chen, M.D, Chair

Institutional Review Board

Peking Union Medical College Hospital

Chinese Academy of Medical Sciences
